# Spurious passage of *Calodium hepaticum* in human stools misidentified as *Trichuris* spp. infection in school-age children in Angola

**DOI:** 10.1128/jcm.01026-25

**Published:** 2026-02-09

**Authors:** Sze Fui Hii, Vito Colella, Adam W. Bartlett, Marta Sólveig Palmeirim, Lucas G. Huggins, Elsa Mendes, Susana Vaz Nery, Rebecca Traub

**Affiliations:** 1 Melbourne Veterinary School, Faculty of Science, The University of Melbourne 98487, Parkville, Victoria, Australia; 2 Kirby Institute, University of New South Wales, Sydney, Australia; 3 Ministry of Health 613780 , Luanda, Angola; 4 School of Veterinary Medicine, Murdoch University 5673, Murdoch, Western Australia, Australia; Mayo Clinic Minnesota, Rochester, Minnesota, USA

**Keywords:** Zoonosis, Rats, *Capillaria*, molecular epidemiology, diagnostics, soil-transmitted helminths, intestinal parasites

## Abstract

*Calodium hepaticum* (syn. *Capillaria hepatica*) is a zoonotic parasite of murid rodents and other mammals, while also being an important agent of hepatic capillariasis in humans. In a cross-sectional survey of schoolchildren across three provinces (Huambo, Uíge, and Zaire) in Angola, we found 39 fecal specimens that were considered positive for *Trichuris trichiura* eggs using the Kato-Katz thick smear but were negative by quantitative polymerase chain reaction (qPCR). Further morphological examination of these putative *Trichuris* eggs identified them as having an asymmetrical bipolar plug morphology, resembling capillariid eggs. Molecular characterization through Sanger sequencing and nanopore metabarcoding confirmed these eggs as *C. hepaticum*, indicating spurious human passage. Once passed in human feces, these eggs may embryonate and become infective in the environment, with the potential to cause hepatic capillariasis in humans. Future research should assess the prevalence of both spurious passage and hepatic capillariasis in humans in Angola, as well as evaluate the impact of misidentification of these nematodes with *Trichuris* eggs on soil-transmitted helminths surveys.

**IMPORTANCE:**

*Calodium hepaticum* causes hepatic capillariasis in humans living in low- to upper-middle income countries. This disease is mainly caused by ingestion of embryonated eggs, whereas consumption of eggs that are unembryonated, such as those found in the livers of infected animals, leads to spurious passage. Notably, unembryonated eggs are morphologically similar to those of *T. trichiura*, leading to potential misdiagnosis in routine fecal examinations and inaccurate estimates of soil-transmitted helminth prevalence. In this study, we detected *C. hepaticum* eggs in fecal specimens initially identified as positive for *T. trichiura* eggs by the Kato-Katz thick smear but tested negative by qPCR. Our findings highlight diagnostic challenges posed by such morphological similarities. Additionally, the detection of *C. hepaticum* eggs in fecal specimens from school-aged children suggests a potential risk of hepatic capillariasis in this population, underscoring the importance for local health authorities and medical practitioners in Angola to consider this parasite as a potential differential diagnosis in cases of unexplained hepatic dysfunction.

## INTRODUCTION

Soil-transmitted helminths (STH) affect an estimated 1.5 billion people worldwide, particularly those living in low- and middle-income countries or in communities with poor access to clean water, sanitation, and hygiene (1). These helminths include hookworms (*Necator americanus*, *Ancylostoma ceylanicum*, and *Ancylostoma duodenale*), *Ascaris lumbricoides*, whipworms (*T. trichiura*), and *Strongyloides stercoralis*. STH infections can cause malnutrition, anemia, and stunting (2). Children, pregnant and lactating women, and agricultural workers living in endemic areas are most at risk for developing severe symptoms and pathology due to STH infections (1).

The Kato-Katz thick smear is one of the techniques recommended by the World Health Organization (WHO) for field diagnosis of STHs due to its ease of use in the field and capacity to enumerate eggs (3, 4). However, the Kato-Katz and other coproscopic techniques are less sensitive and specific when compared to molecular diagnostic tools (5, 6). Morphological misidentification is common due to similarities in egg appearance between different helminth species and due to the presence of artifacts (6). For instance, *Meloidogyne* (root-knot nematodes) can be misidentified as hookworm eggs (6), pollen grains as *Ascaris* eggs (7), and *Calodium hepaticum* eggs as *T. trichiura* in humans (4). These inaccurate diagnoses can lead to incorrect prevalence estimates of infection prevalence and intensity.

*Calodium hepaticum* is a zoonotic nematode belonging to the subfamily Capillarinae of the family Trichuridae (8). This nematode parasitizes liver tissues of murid rodents, the primary definitive host, and a wide range of other mammals including humans (9).

**Fig 1:**
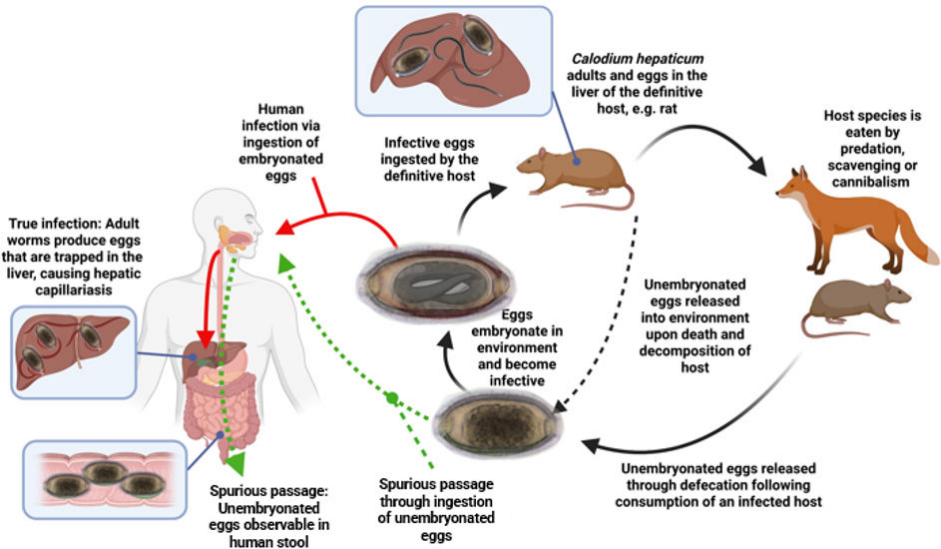
The life cycle of *Calodium hepaticum* and mechanism through which humans can obtain true infections or spurious passage of the parasite.

This parasite causes two types of infections in humans: hepatic capillariasis (true infection) and spurious passage (10). Hepatic capillariasis occurs through ingestion of embryonated eggs that contaminate soil, food, and water. Once ingested, larvae hatch in the intestine and migrate to the liver where they develop into adults and reproduce (Fig. 1) (10). Fertilized but unembryonated eggs produced by female worms remain trapped in the liver and cannot be passed in the feces of the host. In humans, the worms and deposition of eggs in the liver parenchyma cause granuloma formation and liver necrosis (11). Symptoms of hepatic capillariasis include abdominal pain, weight loss, fever, chills, hepatomegaly, ascites, and hepatolithiasis (11). Diagnosis of hepatic capillariasis involves a combination of clinical assessment, laboratory investigations, serology, liver biopsy, and imaging studies (11). The trapped eggs and adult worms of *C. hepaticum* cannot be passed in the host’s feces; therefore, the diagnosis of hepatic capillariasis cannot be made through fecal examination and remains challenging in humans.

Spurious passage in humans occurs when unembryonated eggs present in the infected livers of animals or in the environment are ingested and mechanically passed through the gastrointestinal tract into the feces (11). Animals may also require spurious passage through predation, scavenging, and cannibalism (Fig. 1). Once excreted into the environment, eggs embryonate under suitable conditions of temperature and humidity. Additionally, eggs may also be released into the environment when infected animals die and their carcasses decompose. Unembryonated eggs require 5 to 8 weeks to develop into embryonated forms (12). Once embryonated, eggs become infective, and upon ingestion, they can lead to hepatic capillariasis (Fig. 1) (11, 13–15).

In a 2021 cross-sectional survey investigating the prevalence and infection intensity of STHs in school-aged children in Angola (16), multiple fecal specimens were considered positive for *Trichuris* eggs using the Kato-Katz thick smear method but were negative by qPCR. Given the potential for misidentification of capillariid eggs with those of *T. trichiura*, the present study aimed to clarify the identity of these infections by *Trichuris*-like parasites within these specimens and improve our understanding of STH epidemiology and control requirements in Angola.

## MATERIALS AND METHODS

### Collection of fecal specimens and STH diagnosis

A cross-sectional survey of 3,064 schoolchildren across 219 schools in three provinces (Huambo, Uíge, and Zaire) of Angola was conducted to assess the impact of a 2014–2020 school-based preventive chemotherapy program for schistosomiasis and STH infections (16). Fecal specimens were collected from May to August 2021 and analyzed using the Kato-Katz thick smear and multiplex qPCRs for the detection of *Strongyloides* spp., *Ascaris* spp., *Trichuris* spp., *N. americanus*, *A. ceylanicum,* and *A. duodenale* (16).

Overall, qPCR demonstrated higher sensitivity than Kato-Katz for detecting *T. trichiura* (67.3% vs 61.5%) (16). However, discrepancies were observed, as several specimens that were considered positive for *Trichuris* eggs by the Kato-Katz were negative by qPCR, including some with egg counts exceeding 7,000 eggs per gram (EPG). To investigate these inconsistencies, microscopic examination was repeated on these specimens with high *Trichuris-*like egg intensities using a direct simple wet smear under CX43 Biological Microscope, Olympus Life Sciences (Japan) (×100, ×200, and ×400 magnifications). The length and width of these eggs were measured using Olympus cellSens Entry software version 3.2 (Nagano, Japan), and they were found to be consistent with capillariid eggs according to a previously published study (4).

### Molecular characterization of capillariid eggs

All DNA specimens extracted from Kato-Katz positive but qPCR negative feces (*n* = 39) were subjected to a novel nested PCR targeting a partial region (307 bp) of 18S ribosomal RNA (rRNA) gene of Capillariidae using newly designed primary (Cap18SRT-F1: 5′-GACTGGCTGCTTTGCGCAGT-3′ and Cap18SRT-R1: 5′-CAATCCAACTACGAGCGGTTC-3′) and secondary primers (Cap18SRT-F2: 5′-ATCGCACGGTCCTAGTACCG-3′ and Cap18SRT-R2: 5′-GCTGCTGGCACCAGACTTGC-3′). Primary PCR amplification was performed in a 25 μL reaction mixture containing 2 μL of DNA, 5 μL 5× PCR buffer, 200 μmol/L dNTP, 2.0 mmol/L MgCl_2_, 0.5 units of GoTaq polymerase (Promega, Madison, WI, USA), and 10 pmol of each forward and reverse primer made to the final volume with nuclease free water. PCR cycling conditions comprised an initial activation step at 95°C for 5 min, followed by 40 cycles of 95°C for 30 s, 60°C for 30 s, and 72°C for 30 s with a final extension step of 72°C for 5 min. The secondary round of PCR amplification was performed as for the primary round except that the annealing temperature was set at 62°C. Since the nested PCR could not distinguish between species within the family Capillariidae, all 39 specimens were also subjected to a previously published PCR targeting a 620 bp region of 18S rRNA gene of Trichuridae nematodes (17).

Amplified product was examined on 1.5% agarose gels stained with GelRed nucleic acid stain (Biotium, USA) run at 100 V for 40 min and visualized using UV transillumination. Positive PCR products were submitted to Macrogen Ltd (Seoul, South Korea) for sequencing. DNA sequences were analyzed using Finch TV 1.4.0 (Geospiza Inc.) and compared with those available in GenBank’s nucleotide database using the BLAST algorithm (BLAST Basic Local Alignment Search Tool, 2023).

### Nanopore sequencing and phylogenetic analysis

Nanopore sequencing was also conducted to obtain a larger and more taxonomically informative barcoding region and further aid in the genetic characterization of the eggs using the “Nemabiome” pipeline developed by Huggins et al. (18). Four fecal DNA specimens that were positive for *C. hepaticum* in both PCRs above were subjected to nanopore sequencing on the MinION Mk1B platform (Oxford Nanopore Technologies, Oxford, UK), using the PCR Barcoding Expansion 1-96 (EXP-PBC096) with Ligation Sequencing Kits (SQK-LSK110). The laboratory and bioinformatic pipeline carried out was the same as that reported in Huggins et al. (19, 20) with the only adaptation being the use of the nematode-specific primers Nem_18S_R reverse complemented to be used as a forward primer (21) and NC5 reverse complemented to be used as a reverse primer (22). These primers generate an amplicon of ~750 bp.

For phylogenetic tree construction, DNA sequences were analyzed using Finch TV 1.4.0 (Geospiza, Inc.) and aligned using BioEdit version 7.2.6.1 with the 18S rRNA gene of various Trichuridae species sourced from GenBank. Neighbor-joining method analyses were conducted with Kimura two-parameter model, and trees were constructed using Mega 11 version 11.0.13 (https://www.megasoftware.net/). Bootstrap analyses were conducted using 5,000 replicates.

For Bayesian phylogenetic inference (BI), FASTA alignments were converted to NEXUS format with BI trees inferred using MrBayes 3.2.7 (23). Each BI was performed with two million Markov Chain Monte Carlo (MCMC) generations, sampling every 100th generation with four chains by allowing for transitions and transversions with gamma-distributed rates. Trees outputted by MrBayes were imported into FigTree (version 1.4.4) and then Adobe Illustrator version 27.3.1 (Adobe, San Jose, USA) for editing and merging of bootstrap and posterior probability values, as well as to improve clarity.

## RESULTS

Discrepancies between *Trichuris* qPCR and Kato-Katz were observed in 39 specimens (Fig. S1 and Table S1; Supplemental information). Of the 39 specimens, 22 were from Huambo, 9 from Uíge, and 8 from Zaire provinces in Angola. The intensity of *Trichuris*-like infection for these specimens by Kato-Katz ranged from 12 to 157,560 EPG, with 4 specimens containing more than 7,000 EPG.

**Fig 2:**
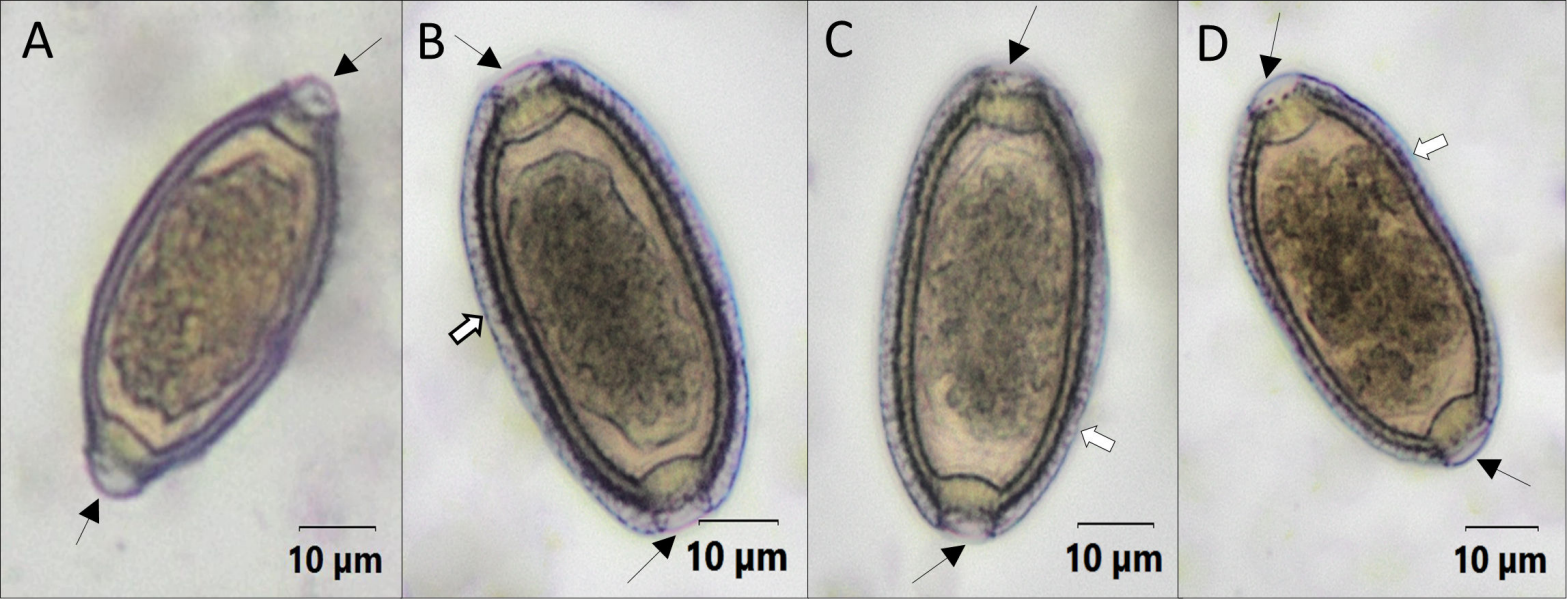
*Trichuris* and capillariid eggs found in fecal specimens from schoolchildren in Angola. (**A**) *Trichuris* eggs are symmetrical and barrel shaped with protruding plugs (black arrows). (**B–D**) Capillariid eggs (shown here: *Calodium hepaticum*) are characterized by smooth bipolar plugs (black arrows), thick striated shell (white arrows), and asymmetrical barrel shape.

Microscopic examination was repeated on 23 of these specimens (*n* = 39). Among these, 10 specimens were found to contain capillariid eggs. These eggs (*n* = 30) had a mean length of 54.32 ± 1.94 µm and width of 29.22 ± 1.28 µm. These eggs had a thick striated shell and were more ellipsoid and asymmetrical compared to the barrel-shaped *Trichuris* egg (Fig. 2). They also had smooth bipolar plugs as opposed to protruding ones possessed by *Trichuris* (Fig. 2). The morphology and morphometry of these eggs were consistent with *C. hepaticum* eggs.

A portion of the 18S rRNA gene was successfully amplified and sequenced from 14 specimens (11 from Huambo and 3 from Uíge) using the newly designed nested PCR. This included all 10 specimens that tested positive for capillariid eggs by microscopy. Direct alignment of the partial 18S rRNA gene sequences revealed 100% nucleotide identity with *C. hepaticum* (GenBank: LC425008), *Pearsonema* spp. (GenBank: LC052387) and *Aonchotheca paranalis* (GenBank: MF621021).

Five DNA sequences of the longer (~620 bp) portion of the 18S rRNA gene were 100% identical to *C. hepaticum* (GenBank: MG686613), 99.35% to *Pearsonema plica* (GenBank: MF621034), and 99.03% to *Aonchotheca putorii* (GenBank: LC052361). These sequences were deposited in GenBank (accession no. OR135793–OR135797). In one specimen, the presence of two PCR products of similar size led to overlapping sequence signals, resulting in a sequence with only 84% identity to *C. hepaticum*. All these six specimens were also positive by the novel nested PCR.

Four of the six specimens that were positive in both PCRs, including one with ambiguous sequencing results, were re-analyzed by nanopore sequencing. A total of 71,136 (specimen AH58-18), 129,199 (specimen AH07-17), 171,609 (specimen HH17-04), and 44,417 (specimen O06-24) reads were obtained. After quality filtering and classification, a total of 66,524 (AH58-18), 99,768 (AH07-17), 98,207 (HH17-04), 34,785 (O06-24) reads were classified as *C. hepaticum*. The dominant *C. hepaticum* sequences from all four specimens were identical and obtained a top hit of 99.74% identity and 100% query cover to *C. hepaticum* in GenBank (LC425008). These four sequences were deposited in GenBank (accession no. PV225280–PV225283).

**Fig 3:**
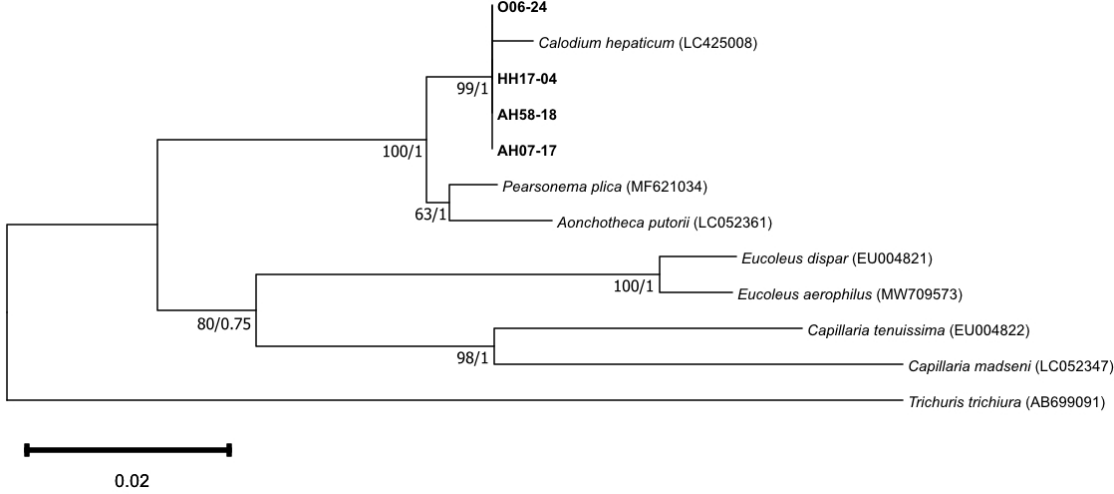
Phylogenetic relationship of *Calodium hepaticum* sequences (bold) identified from the feces of four individuals from Angola alongside representative sequences from across the Trichuridae. Phylogenetic inference was made using a neighbor-joining distance and Bayesian method for a 759 bp segment of the 18S ribosomal RNA gene. The *C. hepaticum* sequences detected in this study clustered with *C. hepaticum* identified in *Rattus norvegicus* in Indonesia (GenBank: LC425008). Bootstrap support and posterior probability values (where available) for tree branches are indicated, with *T. trichiura* used as an outgroup.

Next, these four nanopore sequences were used for phylogenetic analysis of a 759 bp region of the 18S rRNA gene (Fig. 3) identified them all as grouping closely with a *C. hepaticum* sequence obtained from Indonesian *Rattus norvegicus* (GenBank: LC425008).

## DISCUSSION

In this study, we morphologically and genetically characterized *C. hepaticum* eggs causing spurious passage in human stools in Angola that were initially misidentified as *T. trichiura* infections through Kato-Katz.

In Africa, hepatic capillariasis in humans, rodents, and other mammals has been reported in the Côte d'Ivoire, Ethiopia, the Democratic Republic of the Congo, and South Africa (9, 12, 24–26). The source of spurious passage in the present study is unknown but could be associated with the customary habits of ingesting the viscera of wild mammals, as reported elsewhere (10, 27).

In the current study, the presence of *C. hepaticum* eggs in feces is not necessarily associated with hepatic capillariasis. In areas where unsanitary and poor hygienic conditions exist, these unembryonated eggs if released into the environment could potentially embryonate to infective stages and be transmitted to humans via the ingestion of contaminated soil, water, and fresh produce, leading to an increased risk of hepatic capillariasis (11). Similarly, the existence of humans with spurious passage in these three regions in Angola means that they live in an environment potentially contaminated with infective eggs and, as such, are at risk of developing hepatic capillariasis. Humans in close contact with rodents and children below the age of 8 years are reported to be the most affected group due to increased exposure through pica (11).

The presence of hepatic capillariasis in the studied communities remains unclear. When diagnosed, hepatic capillariasis is primarily treated with antihelminthic drugs such as albendazole, thiabendazole, and mebendazole for at least 20 days (11). Even though the WHO recommends mass drug administration of a single dose of albendazole and mebendazole for STHs in school-aged children (2), it is unlikely such a single-dose is effective in controlling hepatic capillariasis. Hence, the best strategies to prevent hepatic capillariasis are good personal hygiene, safe food practices, rodent control, and proper sanitation.

Our results demonstrate a key limitation of the Kato-Katz method, i.e., difficulties with being able to accurately and reliably distinguish *Trichuris* from capillariid eggs. Limited expertise, combined with the large number of specimens that require processing for parasite egg identification during large-scale surveys, can lead to the morphological misidentification of capillariid eggs as those of *Trichuris*. False positives in the diagnosis of *Trichuris* infections may lead to inaccurate prevalence and intensity data for *Trichuris* infections, which could lead to inappropriate monitoring and evaluation of intervention programs.

Our results demonstrate the high specificity of qPCR for *T. trichiura* and its value in monitoring its prevalence, as well as its utility in overcoming the limitations of coproscopic examination. Training of laboratory technicians to differentiate *Trichuris* from capillariid eggs is essential to minimize false positive results.

Limitations of this study include the fact that only discrepant specimens that were positive for *C. hepaticum* eggs via Kato-Katz thick smear and negative for *Trichuris* spp. by qPCR were subjected to conventional PCR. Therefore, the number of spurious passages of *C. hepaticum* is likely underestimated in this study population. The detection of *C. hepaticum* in feces was an incidental finding; therefore, hepatic capillariasis was not specifically assessed in the study, and the true extent of infection remains unknown. The nested PCR designed in this study lacked specificity for *C. hepaticum* and was unable to differentiate between species within the family Capillariidae, including *C. hepaticum*, *Pearsonema* spp., and *Aonchotheca paranalis*. The second PCR protocol used in this study (17) had been validated on isolated capillariid worms and eggs only. Therefore, the low sensitivity and specificity found in this study can be attributed to a lack of diagnostic validation of that protocol using clinical specimens. Here, we employed a novel nemabiome pipeline (18) which demonstrated its effectiveness in rapidly identifying cryptic and/or neglected nematodes with unprecedented precision.

### Conclusion

In this study, we detected spurious passage of *C. hepaticum* in human stools which were misidentified as *Trichuris* spp. infection in school-age children in Angola. The findings of this study highlight the risk of hepatic capillariasis among school-age children in the country, underscoring the necessity for local health authorities and medical practitioners to remain vigilant regarding this parasite as a potential differential diagnosis for hepatic dysfunction in humans in Angola. Future research should assess the prevalence of both spurious passage and hepatic capillariasis in humans in Angola, as well as the risk of misidentification with *Trichuris* eggs during STH surveys.

## Data Availability

The sequences in the study were uploaded to GenBank (accession no. OR135793–OR135797 and PV225280–PV225283).

## SUPPLEMENTAL MATERIAL

Figure S1 jcm.01026-25-s0001.docx The sequential workflow used for detecting *C. hepaticum*.

Table S1 jcm.01026-25-s0002.xlsx Detection of Calodium hepaticum in human fecal samples collected in Angola.

ASM does not own the copyrights to Supplemental Material that may be linked to, or accessed through, an article. The authors have granted ASM a non-exclusive, world-wide license to publish the Supplemental Material files. Please contact the corresponding author directly for reuse.
