# Supplementary figures and images for: Spurious passage of Calodium hepaticum in human stools misidentified as Trichuris spp. infection in school-age children in Angola
Source: J Clin Microbiol. 2026 Feb 9;64(3):e01026-25. doi: 10.1128/jcm.01026-25 (PMC13077770; doi:10.1128/jcm.01026-25)

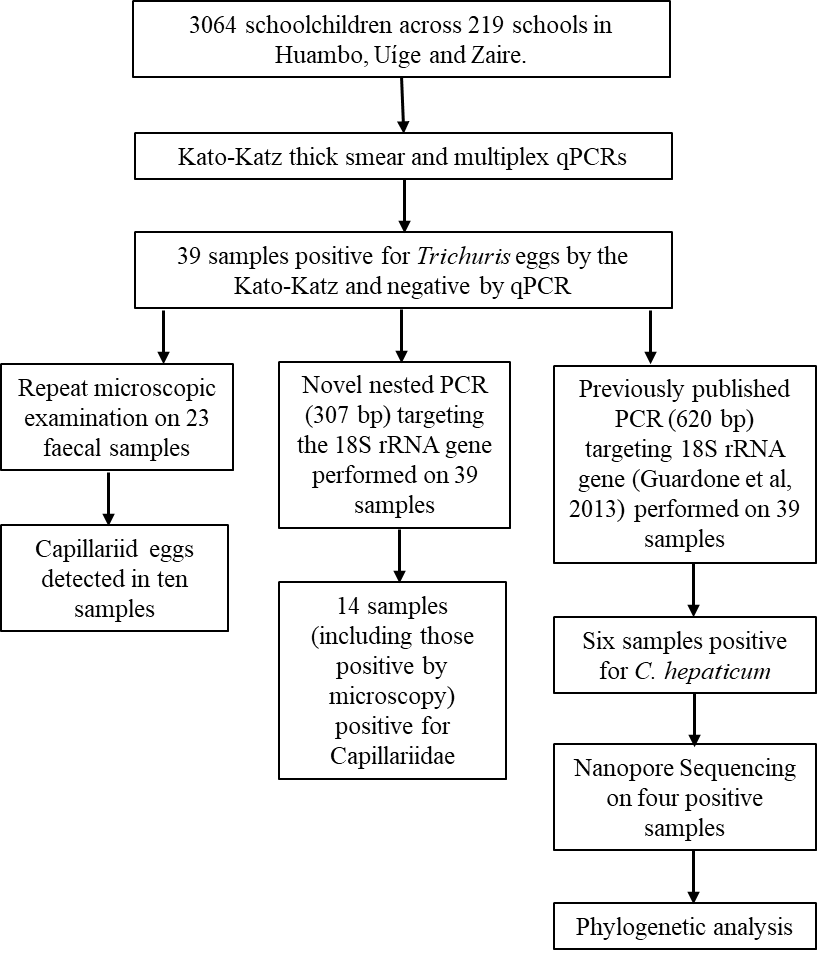


**Figure S1.** The schematic illustrates the sequential workflow used for detecting *C. hepaticum*.

Supplement: Figure S1 — The sequential workflow used for detecting C. hepaticum. [file jcm.01026-25-s0001.docx]
